# Detecting inattentive respondents by machine learning: A generic technique that substitutes for the directed questions scale and compensates for its shortcomings

**DOI:** 10.3758/s13428-024-02407-2

**Published:** 2024-04-08

**Authors:** Koken Ozaki

**Affiliations:** Graduate School of Business Sciences, University of Tsukuba, 3-29-1, Otsuka, Bunkyo-ku, Tokyo, 112-0012 Japan

**Keywords:** Inattentive respondents, Machine learning, The directed questions scale, Web surveys

## Abstract

Web surveys are often used to collect data for psychological research. However, the inclusion of many inattentive respondents can be a problem. Various methods for detecting inattentive respondents have been proposed, most of which require the inclusion of additional items in the survey for detection or the calculation of variables for detection after data collection. This study proposes a method for detecting inattentive respondents in web surveys using machine learning. The method requires only the collection of response time and the inclusion of a Likert scale, eliminating the need to include special detection items in the survey. Based on data from 16 web surveys, a method was developed using predictor variables not included in existing methods. While previous machine learning methods for detecting inattentive respondents can only be applied to the same surveys as the data on which the models were developed, the proposed model is generic and can be applied to any questionnaire as long as response time is available, and a Likert scale is included. In addition, the proposed method showed partially higher accuracy than existing methods.

## Introduction

Web surveys have become a popular data collection tool not only in psychological research but in the social sciences in general. They can be used to produce large amounts of data from respondents of various demographics at a modest cost. Recently, however, the inclusion of inattentive respondents in web surveys has been a serious problem, and more attention is being paid to their detection.

According to Bowling et al. (2016), IER (Insufficient Effort Responding) “occurs when research participants provide inaccurate data because they have failed to carefully read or comply with questionnaire instructions and item content.” IER is sometimes referred to as careless responding (CR) (Meade & Craig, 2012). Curran (2016) refers to these types of responses as “C/IE” responses. Following this convention, in this study, inattentive respondents will be referred to as C/IERs.

### Influence of inattentive responses

When conducting web surveys, it is recommended that C/IERs be detected and excluded from the data before analysis (Maniaci & Rogge, 2014; Meade & Craig, 2012), as their inclusion can negatively affect the results. A number of studies on this topic have appeared in the literature. For example, Credé (2010) showed in a simulation study that even 5% random responses negatively affect correlations. Hamby and Taylor (2016) showed that the presence of C/IERs negatively affects the reliability and validity of a scale. Maniaci and Rogge (2014) showed that the presence of C/IERs decreases statistical power. Woods (2006) showed in simulations that 10% inattentive responses to reverse-worded items, even when the scale is unidimensional in nature, will reject the one-factor model and increase the likelihood of the two-factor model, where each factor corresponds to straight word items and reverse-worded items, respectively. Huang et al. (2015) showed that when the mean of a scale deviates from its midpoint, the presence of C/IERs can increase the correlation between observed variables and increase type 1 error. DeSimone et al. (2018) showed through simulations that random responses decreased inter-item correlations, internal consistency, and the first eigenvalue of the scale. Thus, the presence of inattentive respondents is a major problem affecting the quality of data in web surveys (Bowling & Huang, 2018; Weiner & Dalessio, 2006).

Broadly speaking, there are two ways to deal with C/IERs: prevention and detection. Prevention refers to measures to reduce the number of such respondents. Ward and Meade (2018) showed that surveys devised to increase cognitive dissonance and hypocrisy had fewer C/IERs than controls. Ward and Pond (2015) successfully reduced C/IER by displaying a virtual human to the respondents. However, despite significant efforts at prevention, C/IERs will likely be present in any survey data, making methods of detection highly important.

### Two methods for detecting C/IERs

Two main methods for detecting C/IERs have thus far been considered in the literature: a priori methods, in which items for detection are incorporated into the survey, and post hoc methods, in which indicators for detection are calculated from the collected data. Typical indicators for a priori and post hoc methods are summarized in Table 1.

**Table 1:** Methods for detecting C/IERs

A priori methods	
DQS	The Directed Questions Scale (DQS) uses an item that contains an explicit instruction to those responding to the item, such as “Please select ‘strongly agree’ for this item.” If the instruction is not followed, the respondent is considered a C/IER
IMC	The Instructional Manipulation Check (IMC) (Oppenheimer et al., 2009) uses a Likert scale or multiple-choice question that includes a message in the instruction asking the respondent not to respond in the usual way; that is, the respondent is asked to give a non-intuitive, uncommon response. If the respondent gives the usual response, the respondent is judged to be a C/IER
ARS	The Attentive Responding Scale (ARS) (Maniaci & Rogge, 2014) can be decomposed into an inconsistency subscale and an infrequency subscale. The inconsistency subscale examines the degree of disagreement in responses to pairs of items measuring similar content. The infrequency subscale consists of six items that are considered to be generally answered in a certain way
Bogus items	Bogus items are generally impossible or improbable questions
Self-report items	Asking respondents to self-report their attentiveness/reliability with a question such as, “Should I use your data?”
Response time	Fast responses are likely to occur if the respondent’s purpose is to complete the response quickly and be rewarded as a survey monitor
Post hoc methods	
Invariability	Long string (LS) is the (maximum) number of consecutive responses to questions in the same category on a Likert scale, etc. Intra-individual response variability, or IRV (Dunn et al., 2018; Marjanovic et al., 2015), measures the standard deviation of the respondent’s response results and is used to indicate how little variation there is in the responses. C/IERs tend to respond consecutively to questions in the same category or show less variably in their responses
Outlier analysis	The Mahalanobis distance can be used to assess how anomalous a respondent’s response is compared to the other respondents
Statistically based consistency index	Meade and Craig (2012) formed item pairs that were found a posteriori to be highly correlated from the collected data and proposed a method to examine the degree of disagreement in responses to the item pairs

### Proportion of C/IERs

A priori and post hoc methods provide an indication of the proportion of survey respondents who are C/IERs. Johnson (2005) found that 3.5% of respondents repeatedly selected the same response options without reading the item content. Meade and Craig (2012) found that the proportion of C/IERs among all respondents was 11% by a latent profile analysis using response time and the Mahalanobis distance, while Maniaci and Rogge (2014) found that the proportion of C/IERs was 2.5%. Arias et al. (2020) applied a factor mixture model and found that between 4.4% and 10% were C/IERs. Bruhlmann et al. (2020) applied a latent profile analysis and found that 45.9% of the crowdsourced sample were C/IERs. Jones et al. (2022) estimated an average C/IER rate of 11.7% through a meta-analysis of 48 alcohol-related studies using crowdsourcing. Thus, the proportion of C/IERs varies considerably from study to study.

### How to detect C/IERs using various indicators

In order to detect C/IERs using the indicators listed above, appropriate evaluative criteria need to be established. For example, in the case of DQS, the researcher needs to decide how many DQS items to use and how many of these should be considered indicative of a C/IER if not followed. For response times, Bowling et al. (2016) suggested a value of 2 s per item for determining C/IERs. If the survey system is not equipped with a mechanism for directly measuring response time per item, it can be calculated by dividing the response time per survey form or page by the number of items. However, in such cases, the value of 2 s per item may not be appropriate if the items involve a variety of response formats rather than only a Likert scale. For LS, Huang et al. (2012) recommended 6–14 items, but in the absence of reverse items, it may be possible to answer several questions in the same category consecutively.

Thus, although cutoff criteria have been proposed for each indicator, a consensus has not yet been reached on the “best” cutoff values. In addition, individuals who are identified as a C/IER by one method will not necessarily be judged a C/IER by another method (Curran, 2016). Recommendations that the user understand the characteristics of each method and apply them in some combination seem sound, but there is still no consensus on how to integrate each indicator to determine whether a respondent is inattentive. Ward and Meade (2023) state clearly that there are no clear guidelines on how to detect inattentive respondents using the various indicators.

While there are no generally accepted guidelines, Maniaci and Rogge (2014) argue for the effectiveness of DQS and ARS, noting that DQS showed effectiveness in terms of statistical power when either one or three DQS items were used. Ward and Meade (2023) set three screening levels – minimal, moderate, and extensive – and suggested indicators to be used for each stage. They recommend the use of DQS, response time, LS, and Mahalanobis distance. Notably, while researchers have proposed various methods for detecting C/IERs, most cite the usefulness of DQS.

### Negative effects of using DQS, IMC, and bogus items

The impact on survey respondent perceptions resulting from the use of various detection measures and their impact on response results have also been studied. Kung et al. (2018) found that using DQS or IMC did not affect the measurement properties of the scales. On the other hand, Breitsohl and Steidelmüller (2018), using bogus items, DQS, and IMC, found that such attempts to detect C/IERs were perceived by some as insulting and undermined the respondents’ trust in the researcher, and, in some cases, led to more attentive responses. They further showed that the presence of DQS or IMC items negatively affects the goodness of fit for the factor analysis model. Similar to the findings of Breitsohl and Steidelmüller (2018), Oppenheimer et al. (2009) also suggested that the use of IMC may be taken by respondents as an insult and render the researcher untrustworthy in their eyes. In addition, Curran and Hauser (2019) found that even very diligent respondents agreed with some of the impossibilities included in the bogus items. Thus, such bogus items may lead to misclassifying careful respondents as C/IERs. In summary, although the effectiveness of DQS and other inattentiveness detection methods has been recognized by many, some studies have raised the issue of the negative impact of these methods on respondent perceptions and response results. To address this problem, we sought to develop a machine learning method to detect C/IERs without the use of DQS, IMC, or bogus items.

### Previous studies on machine learning methods

**Fig. 1:**
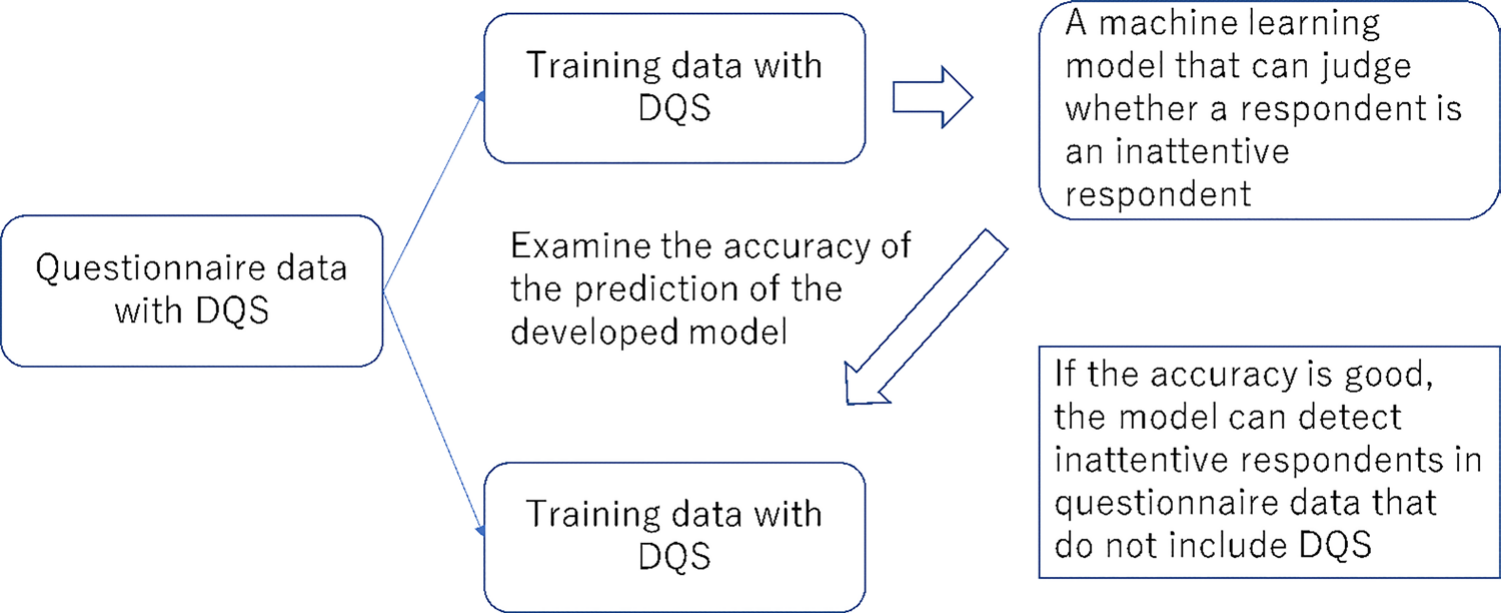
Framework and advantages of detecting inattentive respondents with machine learning models. *Note:* This figure provides a framework for detecting inattentive respondents through machine learning: the survey data, including DQS, are divided into training and test data. Using the training data, a machine learning model is developed to predict the DQS with predictors, and the model is fitted to the test data. If the fit is good, it is possible to detect C/IERs without using DQS for that survey

Supervised machine learning methods for C/IER detection have been proposed in several recent studies (Ozaki & Suzuki, 2019; Gogami et al., 2021; Schroeders et al., 2022). Supervised machine learning is generally used when there is an outcome to predict or detect. In the case of C/IER detection, the C/IERs in the data are identified by a measure such as DQS, and a machine learning model is developed to detect such respondents using a set of predictors typically collected in surveys or calculated from these variables. Once the machine learning model is developed, detection with a certain degree of accuracy is possible so long as there are suitable predictors, making it no longer necessary to use DQS or other a priori methods to identify C/IERs. Figure 1 illustrates this idea.

The contributions of Ozaki and Suzuki (2019), Gogami et al. (2021), and Schroeders et al. (2022) to the use of supervised learning to detect inattentive respondents are described below. The significance of their studies is threefold: (1) As noted, there are possible problems with the use of indicators such as DQS for C/IER detection due to their negative impact on respondent perceptions and the resulting estimates (Breitsohl & Steidelmüller, 2018). This problem can be eliminated if an effective machine learning alternative can be developed; (2) Detection items like DQS are essentially add-on survey items, and thus eliminating them is desirable. A proper machine learning model has the potential to achieve this; (3) Various indicators have been proposed for detecting C/IERs, but it is not yet clear how to integrate and use them. Machine learning is capable of producing a single indicator – the inattentive response probability – using each of the indices. Calculating the inattentive response probability by machine learning can be thought of as a way of integrating the indices. Importantly, it is an easy indicator to use, since respondents can be excluded according to their value.

### Ozaki and Suzuki (2019) and Gogami et al. (2021)

In devising their supervised machine learning approach, Ozaki and Suzuki (2019) used a Japanese web research company to conduct a survey to examine the impact of three generations living together on the number of children in a family. They included two DQS items and three item pairs to check for inconsistent responses as outcomes in the survey. The respondents were considered C/IERs if they responded improperly to any one of the five items. Gogami et al. (2021) conducted a crowdsourced survey that included Likert scale items. They also included a three-item DQS and ARS. Respondents were considered a C/IER if they violated any one of the three DQS items or if they had values above the cutoff point on the ARS. Both studies attempted to detect C/IERs identified by DQS and other items using response time, LS, Mahalanobis distance, etc.

The sample used by Ozaki and Suzuki (2019) consisted of 2000 PC respondents (610 C/IERs and 1390 attentive respondents). The data for half the respondents were used as training data (500 C/IERs and 500 attentive respondents). Accuracy was measured by fitting the model developed with the training data to the test data for the remaining 1000 respondents. Gogami et al. (2021) used a sample size of 4940 smartphone respondents (247 C/IERs and 4693 attentive respondents). They randomly selected 247 respondents from the 4693 attentive respondents five times. The accuracy of their models was evaluated using Leave-One-Out Cross-Validation for each C/IER: attentive = 247:247 and calculating the average of the five accuracy evaluation results.

Ozaki and Suzuki (2019) applied various methods, including random forests (Breiman, 2001) and gradient boosting (Chen & Guestrin, 2016; Friedman, 2001, 2002), and used LS for Likert scale items, response time for the entire questionnaire, Mahalanobis distance calculated from the Likert scale items, the *p* value of the Mahalanobis distance, and the gender and age of respondents as predictors. The results showed an accuracy of 81%, a precision of 32%, a recall of 66%, a balanced accuracy of 74%, and a specificity of 83% when the inattentive respondent probability (IRP) obtained by gradient boosting was .5 or higher. Although the precision was low, the proportion of C/IERs present in the test data was reduced by 56% when the respondents who were detected as C/IERs by this method were removed. Gogami et al. (2021) measured and used as predictors the number of times text was deleted and the respondent’s scrolling speed, etc. from smartphone screen operation data. In addition, response time was measured separately for Likert scales and free descriptions and used as a predictor. The number of letters in the open-ended responses, the number of intermediate responses on the scale, and LS also served as predictors. The detection results using gradient boosting showed an accuracy, precision, and recall of approximately 86% (it was not possible to calculate the balanced accuracy and the specificity from the information in their paper), suggesting the effectiveness of using smartphone screen operation data for response data.

It should be noted that, since the training and test data in both studies were from the same survey, it remains unclear as to whether the models that were developed could be used for other surveys. Unless the method is general enough to be applied to other surveys, it cannot be considered practical.

### Schroeders et al. (2022)

Schroeders et al. (2022) used gradient boosting as a machine learning method and conducted a simulation study and a study using real data. In the real data study, the sample was divided into an attentive respondent group and a C/IER group. The attentive group was given the usual instructions, such as taking time to carefully consider the contents of the items, while the C/IER group was asked to respond quickly without carefully reading the contents of the items. The sample size was 605 (244 C/IERs and 361 attentive respondents). From this sample, 226 C/IERs and 199 attentive respondents were randomly selected as training data. The remaining 180 were used as test data. The training and test data were randomly selected 1000 times, and the average accuracy was reported.

In the real data study, a comparison of detection accuracy was conducted in which machine learning (gradient boosting) methods and traditional methods such as the Mahalanobis distance and LS. The predictors for machine learning were the same traditional measures. In addition, the response time was used in the real data study.

While the results of the simulation study involving the machine learning model were generally good, the real data study did not achieve the same degree of accuracy. In the case of gradient boosting, recall was 60%, meaning that the percentage of correctly detected C/IERs was only 60%. Precision was low, at 19%, which means that only 19% of the respondents who were judged C/IERs were actually C/IERs. Recall values for the conventional methods were lower than for machine learning, and precision was less than or equal to that for machine learning. They also reported an accuracy of 70%, a balanced accuracy of 66%, and a specificity of 71%.

Schroeders et al. (2022), while pointing to the possibility that some respondents in the attentive group responded inattentively as a reason why the results of the real data analysis were not favorable, stated that the inattentive response process in the real world is much more heterogeneous than in the simulation and that larger training data sets are needed. They also argue that “generalizations to other data sets, samples, and situations are *not* possible, because every examination is highly specific in terms of items and persons” (Schroeders et al., 2022, p. 49). Our study aimed to challenge this argument and develop a generic method that can be applied to any survey so long as it includes Likert scale items, and response times are available.

## Purpose of this study

A common issue among the three machine learning studies described above is that the methods developed are not generic, meaning that they can only be applied to the questionnaires used to develop them.

The purpose of this study is to offer a method for detecting C/IERs using machine learning that can be applied to any questionnaire. Although the proposed method is not technically generic since it is premised on the condition that Likert scales be included in any survey to which it is applied, this condition is quite modest, as most psychological research includes Likert scales. Another condition is that response times are available, which is the case with most web-based survey systems. Thus, the method can be said to be applicable to most psychological research.

### Advantages, characteristics, and novelty of method developed in this study

Using data from 16 web surveys, a method to detect C/IERs by machine learning for PC and smartphone responses, respectively, was developed. The proposed method has the following six advantages, features, and novelties over existing a priori, post hoc, and machine learning methods:

1. The method developed by Ozaki and Suzuki (2019), Gogami et al. (2021), and Schroeders et al. (2022) uses only one web survey data set; thus, it can only be applied to surveys with the same content as the survey used to develop the model. The method developed in this study is a general-purpose method that can be applied to any survey that includes a Likert scale.
2. Since the deletion of respondents can be done based on the probability of inattentive responses obtained by machine learning, it is unnecessary to comprehensively consider multiple indicators. It is also unnecessary to set a criterion that matches the content of each new survey (although it is necessary to decide what value should be used as the cutoff point for the IRP).
3. Since there is no need to include a DQS item or any other mechanism in the questionnaire in advance, there is no need to be concerned about offending respondents. In addition, eliminating the need to incorporate a DQS item or other detection indicator reduces the number of items, thereby reducing both the burden on respondents and survey costs.
4. The proposed method improves detection accuracy by using new predictors not used in previous studies.
5. By using much more training data than in the three previous studies, a generic model is developed.
6. The layout of the response screen for PCs differs from that of smartphones, as is the way that respondents answer the questions. However, previous studies comparing the differences between PC and smartphone responses indicate that, in general, there is no significant difference in the results for the two types of responses (Tourangeau et al., 2017; Andreadis, 2015). On the other hand, some studies have shown that the response time is longer for smartphone responses (Andreadis, 2015; de Bruijne & Wijnant, 2013; Keusch & Yan, 2017). Since the sample size used in this study is very large, separate models are developed for PC and smartphone responses to achieve more accurate results.

### Method

Sixteen web surveys were used in this study to develop the machine learning models for C/IER detection. A separate model was developed for PC responses and smartphone responses. Since three of the 16 surveys did not produce sufficient PC response data, only 13 surveys were used to develop and test the PC model. This study confirmed advantages 1 through 6 listed above. This study was not preregistered.

Although deep learning (Urban & Gates, 2021) has attracted considerable attention in the field of machine learning, Grinsztajn et al. (2022) showed that tree-based methods are more effective than deep learning when the sample size is less than 10,000. Since the sample sizes in this study are 5610 for smartphone responses and 4704 for PC responses, the detection model was developed using random forests and gradient boosting.

### Summary of 16 web surveys

A summary of the 16 web surveys used in the study is presented in Table 2, which provides information on the survey content, number of items, location of the two DQS items, and the starting position of the Likert scale used to apply the machine learning model. All surveys were conducted between 2020 and 2021 and were conducted primarily by researchers other than the first author of this paper for academic research in psychology or other fields. Therefore, the data were collected in practical situations where the machine learning models to be developed were applied. Of the 16 surveys, nine were for psychological research. Of the remaining seven, three were for business management, three were for marketing, and one was a behavioral study (i.e., behavior after returning home as it relates to COVID-19). Some of the surveys were pre-screened and administered to specific target groups, while others were not targeted. Therefore, diverse data were collected in terms of survey content and survey subjects. Furthermore, as described below, the C/IER rate also varied.

**Table 2:** Information regarding the 16 surveys

ID	Questionnaire Contents	Limitations	Items	Locations	Categories	DQS1	DQS2	Inclusion
1	Respondent’s behavior at work	Regularly employed in a Japanese company with more than 300 employees, not in a managerial position, working in a team of more than 4 and fewer than 15 people, and under 27 years old	119	76	6	30	101	No
2	Respondent’s activities after returning home	20 to 70 years old and living in a residence other than a dormitory or a rooming house	277	247	7	255	271	Yes
3	Current situation of promotions and attitudes toward promotions	A non-managerial employee of the organization	84	11	5	21	62	Yes
4	Daily reactions to positive events	None	78	8	7	30	65	No
5	Effect of self-discrepancy on adjustment	Respondents in their 20s or 30s who responded to the preceding wave 1 survey	100	51	5	14	62	Yes
6	Respondent’s empathy for those close to him/her	20s or 30s	104	8	5	16	53	Yes
7	Socializing	None	112	61	4	36	88	No
8	Respondent’s personality and behavior in the workplace	None	127	6	5	72	90	No
9	Smartphone use and interpersonal relationships	Persons who have used a smartphone	123	101	4	46	78	No
10	Interpersonal relationships (without consent statement)	None	75	8	5	31	61	No
11	Respondent’s travel experiences	Those who have taken a self-organized domestic trip of one night or more within the past 2 years, considered Akita Prefecture as a potential destination, and were living in Hokkaido, Tohoku, Hokuriku, or Kanto Koshinetsu at the time of the trip.	131	99	7	43	75	No
12	Interpersonal relationships	None	75	49	5	32	62	No
13	Sponsorship	Respondents who answered "yes" to both questions, "Have you ever heard of the brand Under Armour?" and "Have you ever bought Under Armour products?"	178	78	7	75	170	No
14	Support from temporary staffing agencies, etc., and changes in attitudes toward work	Temporary workers over 20 years old	88	69	5	29	61	No
15	Respondents and their interpersonal relationships	Students between 18 and 29 years old	152	20	5	11	136	No
16	Theme parks	Those who have visited Tokyo Disneyland or Universal Studios Japan within the last 3 years	109	7	7	53	92	No

"ID" is the survey ID number, "Questionnaire content" is the theme of the survey, "Limitations" is the composition of the surveyed group, "Items" is the number of items, "Location" is the location of 12 items, "Categories" is the number of categories in each Likert scale, "DQS1" is the DQS1 position, "DQS2" is the DQS2 position, and "Inclusion" is whether the 12 items include DQS

Each of the surveys contained Likert scale items, including the two DQS items. The content of the DQS items was similar to “Please select category 2 for this question.” All surveys were approved by the research ethics review committee of the first author’s institution and were conducted for psychology and other research as well as for this study. The first author’s only involvement in the questionnaire design was placement of the DQS items. In order to avoid the noticeable presence of DQS items, placement at the beginning or end of a block of Likert scale items or at the beginning or end of a page was avoided. In the DQS items, respondents were directed to respond in a category other than the middle category because it has been found that Asians, including Japanese respondents, tend to respond in the middle category (Harzing, 2006; Masuda et al., 2017). The positions of the two DQS items are shown in Table 2. The position refers to the column number for the data. The number of items is the total number of columns. All surveys were conducted by the same web research company in Japan. The survey targets were monitors who were registered with the survey company. Although nationalities were not tabulated, it is assumed that most of the registered monitors are Japanese since the language of the questionnaire was Japanese. In addition, as described below, the machine learning model developed in this study utilizes data from 12 consecutive items with a Likert scale. Table 2 shows the position of the first Likert scale item.

Note that survey 10 is nearly identical to survey 12, with the difference being that survey 12 had the respondents promise to respond seriously at the beginning of the questionnaire. Therefore, it is conceivable that survey 10 and survey 12 could be analyzed without treating them as separate surveys. However, since the two surveys contained multiple Likert scales, different Likert scales could be used to create the predictors for machine learning. Since the inclusion of the two surveys allowed examining the effect of the position of the Likert scale on detection accuracy, it was decided to include both surveys for analysis. Table 3 provides information on the number of respondents, the number of C/IERs, and the rate of C/IERs for PC and smartphone responses, respectively.

**Table 3:** Number of respondents and C/IERs in each survey

ID	*n*	C/IERs	Rate	Smart	C/IERs (smart)	Rate (smart)	PC	C/IERs (PC)	Rate (PC)	Age (PC)	Age (smart)	Female (PC)	Female (smart)	PC data analysis
1	1600	805	.50	961	439	.46	639	366	.57	47.6	41.9	.19	.32	1
2	1600	397	.25	806	187	.23	794	210	.26	53.8	43.4	.32	.56	1
3	1600	634	.40	782	303	.39	818	331	.41	50.0	42.9	.29	.45	1
4	1600	462	.29	786	243	.31	814	219	.27	55.8	45.1	.30	.51	1
5	1300	569	.44	817	326	.40	483	243	.50	33.4	27.5	.44	.72	1
6	1444	683	.47	903	403	.45	541	280	.52	27.4	25.9	.46	.75	1
7	1600	509	.32	778	240	.31	822	269	.33	56.7	38.2	.40	.55	1
8	1600	638	.40	811	346	.43	789	292	.37	51.0	44.0	.20	.32	1
9	1200	439	.37	609	233	.38	591	206	.35	45.6	29.9	.41	.69	1
10	1000	383	.38	496	187	.38	504	196	.39	57.9	33.2	.45	.55	1
11	1200	528	.44	580	270	.47	620	258	.42	52.8	40.5	.27	.39	1
12	1600	571	.36	806	274	.34	794	297	.37	55.9	43.4	.01	.27	1
13	1000	499	.50	566	293	.52	434	206	.48	52.5	41.5	.27	.45	1
14	1000	510	.51	703	348	.50	297	162	.55	47.8	39.8	.50	.61	0
15	1500	438	.29	1411	401	.28	89	37	.42	21.7	20.5	.45	.73	0
16	1000	410	.41	721	289	.40	279	121	.43	46.2	36.0	.38	.66	0

"ID" is the survey ID, "*n*" is the number of respondents, "C/IERs" is the total number of C/IERs, "rate" is the C/IER rate, "smart" is the number of smartphone respondents, "C/IERs (smart)" is the number of C/IERs for smartphone data, "rate (smart)" is the C/IER rate for smartphone data, "PC" is the number of PC respondents, "C/IERs (PC)" is the number of C/IERs (PC), "rate (PC)" is the C/IER rate for PC data, "age (PC)" is the mean age (PC), "age (smart)" is the mean age (smartphone), "Female (PC)" is the female rate (PC), "Female (smart)" is the female rate (smartphone), and "PC data analysis" is whether the data were used for PC data analysis

The analysis was performed by R version 4.1.2 (R Core Team, 2022). The randomForest package (Liaw & Wiener, 2002) was used for the random forest analysis and the xgboost package (Chen et al., 2022) was used for the boosting analysis. The raw data cannot be shared with the public due to research ethics.

### Training data and test data

**Fig. 2:**
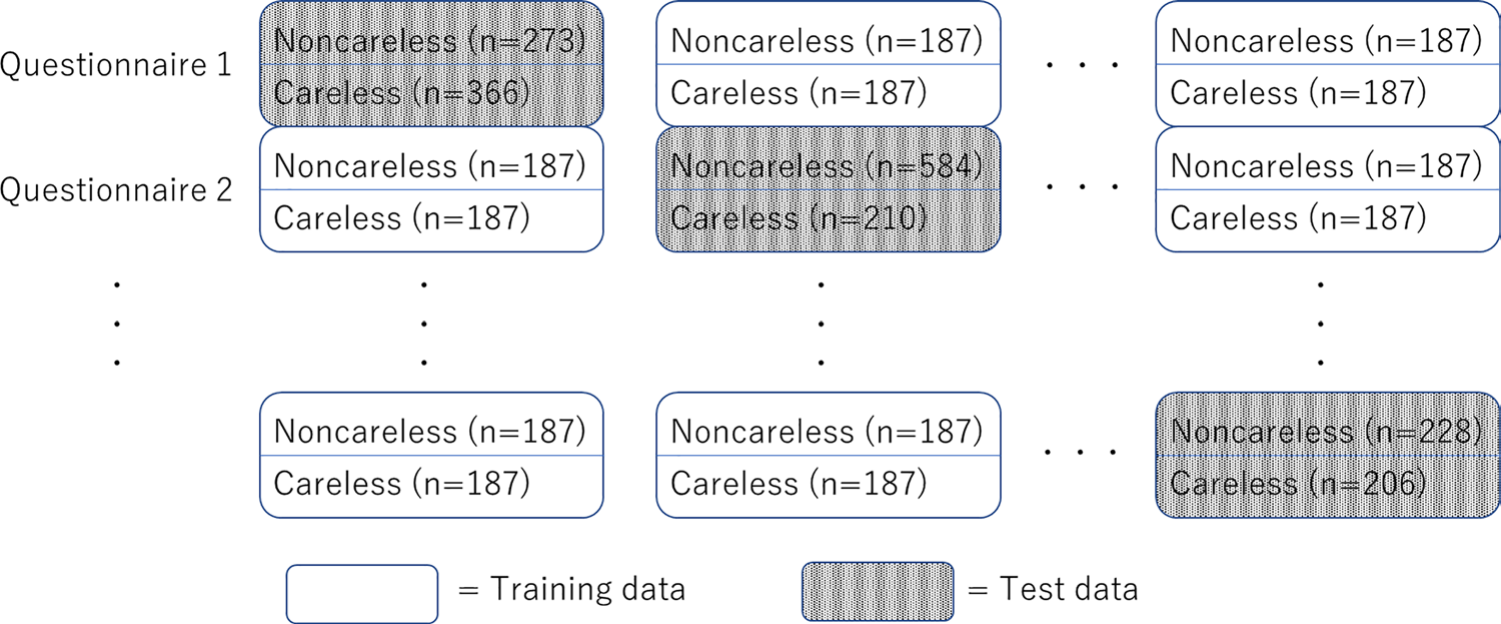
Training and test data for the PC responses. *Note.* This figure shows the development of a machine learning model to detect C/IERs using 13 surveys. In each of the 13 analyses, 12 surveys are used to develop the model. The model is then applied to the one remaining survey to check its predictive accuracy. The same approach is followed for the 16 sets of smartphone response data. See Appendix B for other methods for developing models using multiple surveys

Since the machine learning models were intended to make predictions and detections, the sample data were classified into data for model training and test data for detection by the learned model. In this study, as shown in Fig. 2, out of 13 (16) surveys, 12 (15) surveys were used as training data; the single remaining survey was used as test data to test the detection accuracy of the model developed. The procedure was repeated 13 (16) times. This is an application of Leave-One-Out Cross-Validation, a procedure used to examine the generalization performance of machine learning models.

In terms of detection accuracy, it is desirable for the outcome in the training data to take values of 0 or 1, with each value being the case 50% of the time. For this purpose, rather than using all the data from the surveys, the minimum number of C/IERs for both PC and smartphone responses was used to set the sample size where the outcome in the training data is 1. This same sample size was used for cases where the value of the outcome was 0. For example, for smartphone responses, the minimum number of C/IERs (Table 2) is 187, so the sample size is 2 × 187 = 374 for each survey. As noted earlier, for smartphone responses, 15 surveys were used to develop the model; thus, the total sample size for the smartphone training data is 187 × 2 × 15 = 5610. For the PC responses, the lowest numbers of C/IERs were, in order, 37, 121, 162, and 196. Ultimately, 196 was considered the minimum acceptable number, and the three surveys with fewer than 196 C/IERs were omitted from the analysis. Thus, the total sample size for developing the model for PC responses was 196 × 2 × 12 = 4704. If 162 was used, the total sample size would be 162 × 2 × 12 = 3564, which is obviously smaller; 37 or 121 would also result in a smaller sample size. In the tests conducted to evaluate model performance, all the data in the designated test survey were used.

The sample sizes for the training data in the three previous studies were 1000 for Ozaki and Suzuki (2019), 493 for Gogami et al. (2021), and 425 for Schroeders et al. (2022). Thus, the present study sought to develop a model with high detection accuracy using samples 4.7 to 13.2 times larger than the samples used in the previous studies.

### Outcome

The (1, 0) machine learning outcome in this study indicates whether the respondent responded incorrectly to at least one of the two DQS items included in the survey. If the respondent responded incorrectly to at least one DQS item, the outcome was assigned a value of 1, otherwise 0. Various other indices besides DQS have been used in combination in prior inattentive respondent studies. Nevertheless, in this study, only DQS items were used as a measure of C/IER. There are two reasons for this choice:

1. Ward and Meade (2023) mentioned unambiguity in scoring as an advantage of DQS. Since the goal of supervised learning is to develop a method for predicting the outcome, the outcome should be unambiguous. As noted earlier, Schroeders et al. (2022) recognized the possibility that even respondents who were instructed to be attentive may have given inattentive responses as a reason for the lack of good detection accuracy in their real data study. By using DQS, such a possibility can be ruled out.
2. Maniaci and Rogge (2014) showed that with either three DQS items (where noncompliance with two or more DQS items indicates an inattentive respondent) or one DQS item, the statistical power of the sex difference analysis on the openness factor score was comparable to that of ARS and seven DQS items (where noncompliance with three or more DQS items indicates an inattentive respondent). In addition, Ward and Meade (2023) recommended the use of DQS as a minimal practice to cope with C/IERs. Thus, DQS is often recommended.

As constructed, this study, at the very least, answers the question of whether machine learning can be used as an alternative to DQS. Similar studies might be conducted using other indicators, such as ARS, to flag C/IERs. The results could then be used to answer the question of whether machine learning can be used as a substitute for the other indicators. Such studies are left to future work.

### Predictors

For any of the predictors in the study, it is not possible to compare the magnitude of the values across different questionnaires. For example, since the total response time naturally depends on the length of the questionnaire, it cannot be directly compared across questionnaires. As a result, it is necessary to transform the variables. This section gives the details of the predictors, describes the transformation method, and outlines the reasons for the transformation.

The indicators fall into three main categories: predictors using Likert scales, paradata obtained in the administration of the survey, and predictors using open-ended responses.

### Predictors using Likert scales

Listed below are the study’s predictors using Likert scales. All 16 surveys included Likert scales, although the number of items on the Likert scale varied across surveys. In this study, 12 consecutive Likert items were used to compute the predictors. Costa and McCrae (2008) reported that, of the 983 respondents in their survey, none answered questions in the same category more than 6, 9, 10, 14, and 9 times in a row for each of the five response categories. Based on this and the length of the scale of the questionnaire administered, 12 items were used in the present study. The location of these items within the questionnaire is shown in Table 2. The number of consecutive items can be varied depending on the data to which the machine learning model is applied.

The 12 items were chosen to satisfy one (or both) of the following conditions: they contain reverse-worded items, or they contain items measuring different constructs. A case in which neither of the conditions is met would entail selecting 12 items that measure the same construct and that do not contain reverse-worded items. It is thus possible that predictors such as LS may not work well, since even attentive respondents may choose the same category for 12 consecutive items. Table 2 shows the relationship among the 12 items, indicating that one or both of the above conditions is satisfied in all 16 surveys.

#### LS

LS is defined as the maximum number of consecutive responses to questions in the same category on a 12-item Likert scale. As shown in Table 2, the number of response categories for the scales used to calculate LS differs across surveys; however, we did not standardize the LS to account for the number of response categories. The reason for this is that C/IERs are expected to have a longer LS even if the number of response categories differs. Since all 12 items were used in this study, no standardization by number of items was done; however, when the number of items differs, the rate of LS to the number of items could be used as the predictor “LS/number of items.”

#### Response limited to no more than two categories

Although LS refers to consecutive responses using the same category, it may be possible for a respondent to switch to another category in the middle of responding to questions in the same category in a series or to always choose the adjacent category in a series. Four predictors were used to capture such response behavior (R2C, R3C, AC, and MAC). To the authors’ knowledge, these indicators have not been used before. The first of the four variables, abbreviated as R2C, is “response in no more than two categories,” a binary variable indicating whether the number of categories chosen in response to the Likert scale items used to develop the predictors (in this case, 12 items) is less than or equal to 2. Based on the assumption that C/IERs use a small number of response categories without being affected by the number of categories, no transformation was performed for R2C.

#### Response limited to no more than three categories

Similar to R2C, R3C is a binary variable indicating whether the respondent used three or fewer categories in their responses. R3C is not transformed for the same reasons as R2C.

#### Number of consecutive responses to items in adjacent categories

Variable AC is defined as the total number of consecutive responses in adjacent categories on a Likert scale, staggered by one category. For example, if the respondent responded 1, 2, 3, 4, 2, 1, the value of index AC would be 4 (1-2, 2-3, 3-4, 2-1). In this case, C/IERs cannot be detected by LS, R2C, or R3C. The number of consecutive responses to questions in adjacent categories is abbreviated as AC. AC is standardized as “number of categories*AC,” since the smaller the number of categories, the larger the AC value may be. If the number of items differs, “the number of categories×AC/number of items” is used.

#### Maximum number of consecutive responses to items in adjacent categories

Whereas AC represents the total number of consecutive responses, MAC indicates the maximum number of consecutive responses to questions in adjacent categories. For example, if the responses are 1, 2, 3, 4, 2, 1, the value of this index is 3 (1-2, 2-3, 3-4). The transformation method is the same as that for AC.

Although Dunn et al. (2018) showed the effectiveness of IRV, we chose not to use it in this study. The fact that IRV is a standard deviation makes it difficult to adjust for differences in the number of Likert scale categories across surveys. Instead, R2C, R3C, AC, and MAC were used, because the sum of their ability of each to assess the lack of variability in responses is equal to or better than that of IRV, and because it is relatively easy to adjust for differences in the number of categories. Dunn et al. (2018) stated that IRV can detect cases of responses with small variability that cannot be detected by LS. They then cite examples such as 2, 2, 2, 2, 3, 3, 2, 2, 2 and 4, 5, 4, 5, 4, 5, 4, 5, 5, 5, where adjacent categories are selected one after another. These can be detected by R2C, R3C, AC, or MAC. In addition, responses such as 1, 2, 3, 4, 5, 4, 3, 2 are difficult to detect with IRV, but can be detected with AC and MAC.

#### Mahalanobis distance

The Mahalanobis distance (maha) is an indicator used in various studies. In this study, 12 items were used for the calculation, providing an indicator of how distinctive each respondent is in his/her responses to these 12 items. The Mahalanobis distance maha_i_ for respondent *i* is calculated by the following formula: ${{\text{maha}}}_{{\text{i}}}=\left({x}_{i}{\prime}-{\overline{x} }{\prime}\right){\Sigma }^{-1}\left({x}_{i}-\overline{x }\right)$, where xi is the vector of responses to Likert scale items for respondent *i* and ∑ is the covariance matrix of Likert scale items. It should be noted that the mean vector and covariance matrix needed for the calculation would be incorrect if C/IERs were included in the data. Therefore, the mean vector and covariance matrix used here to compute the Mahalanobis distance were obtained by excluding respondents with LS = 12. The Mahalanobis distance is an index that considers the variance of each variable. Thus, it originally corresponds to the fact that the variance of each item differs from survey to survey due to the different number of response categories in the Likert scale used to create the index. However, because differences in the number of items on the Likert scale affect the Mahalanobis distance, it is transformed by dividing maha by the square of the number of items.

#### *P *value for Mahalanobis distance

A statistical test in which the null hypothesis is “data for the respondent do not deviate from the mean vector” was performed on the maha, using the chi-square distribution with degrees of freedom equal to the number of items. The *p* value in the significance test was then used as another predictor. The *p* value for the maha is abbreviated as “maha_p.”

#### Mahalanobis distance using two variables with highest correlation

The maha using only the two variables with the highest correlation among the pairs of 12 Likert scale items was determined. As with the maha above, the mean vector and covariance matrix were obtained by excluding respondents with LS = 12. The maha using the two variables with the highest correlation is abbreviated as “maha2.”

#### *P *value for Mahalanobis distance using two variables with highest correlation

This is the *p* value for the maha using the two variables with the highest correlation. The number of degrees of freedom is 2. This indicator is abbreviated as “maha2_p.”

#### Sum of absolute values of deviations from mean vector

This index, abbreviated as “absdevi,” is the sum of the absolute values of the differences between the mean vector for the 12 items and each respondent’s responses. The aim of this indicator is similar to that of the maha; however, absdevi does not take into account the covariance between items. Because the value of absdevi increases with the number of Likert scale items and with the number of response categories, it is transformed as “absdevi/(number of items * number of categories).”

### Predictors using paradata obtained from administration of surveys

Auxiliary data collected during the process of administering a survey are called paradata. In this study, the total response time of each respondent and the median of the total response time per survey were used as paradata-based indicators.

#### Total response time

While pointing out that fast responses are one of the characteristics of inattentive respondents, Ward and Meade (2023) also noted that, since there are cases where respondents drop out and come back in the middle of their responses, the response time per page is more accurate for detecting fast responses than the time taken to complete the entire questionnaire. However, the web survey system used in this study lacked a mechanism to measure the response time per page. As a result, the total response time was used. In box-and-whisker plots of the total response time, values greater than the extreme of the upper whisker were considered to be due to the respondent interrupting and then returning to his/her responses. In such cases, the response time for that section was replaced by the extreme of the upper whisker. Similar manipulations were performed by Maniaci and Rogge (2014) and Schroeders et al. (2022).

Since the total response time is affected by the number of items, the total response time divided by the number of items was used to account for differences among surveys. The total response time is abbreviated as “time.”

#### Median of total response time

The median of the above total response time was calculated for each survey and used as a predictor. This is a questionnaire-level variable since it is the same among respondents who took the same survey, although it is a different value for each survey. The median total response time is abbreviated as “time_m.” Since the purpose of this indicator is to express differences among surveys, no transformation by number of items was applied to time_m.

#### Number of survey items

It is assumed that the greater the number of survey items, the more likely it is that inattentive responses will occur. Therefore, the number of survey items was used as a predictor. This, too, is a variable at the questionnaire level. The number of survey items is abbreviated as “nitems.”

The machine learning model was developed using the above 13 variables as predictors. To the author’s knowledge, nine of the 13 variables, R2C, R3C, AC, MAC, maha2, maha2_p, absdevi, time_m, and nitems, are predictors that have not been used in prior studies.

### Parameter tuning

As noted, the model was developed using training data consisting of 4704 (5610) values obtained from 12 (15) surveys. Model parameters were tuned to increase the detection accuracy when the model was applied to the validation data. After constructing the model, the model was fitted to the test data to examine its detection accuracy when applied to data that were not used for model development. The validation data were obtained by bootstrapping from the training data for random forests and by tenfold cross-validation on the training data for gradient boosting.

Random forests constitute a method of generating multiple decision trees and using the averaged tree of these trees for prediction. To generate multiple trees, B different training data are generated from the training data by the bootstrap method, and B trees are generated from the B training data. When generating the trees, the predictors to be candidates for partitioning are also selected by random sampling at each partitioning. The model is fit to the data not extracted in each bootstrap sample (called Out Ob Bug; OOB) to obtain the validation error. The number of predictors sampled in each partition is a parameter determined by searching for the value that minimizes the validation error. Similar to random forests, boosting is a method that uses a large number of forecasting models with decision trees, but differs in that the trees grow sequentially in steps. The depth of the largest tree is a parameter determined by ten-part cross-validation.

### Threshold for inattentive response probability

In this study, the machine learning output is the probability that a respondent will be judged as a C/IER. Given that the output is a probability, it would seem reasonable to differentiate C/IERs and attentive respondents based on a value of .5. Results for criteria other than .5 can be downloaded from https://osf.io/2t64w. A higher threshold means that only respondents who show a high tendency to inattentive responses are judged as C/IERs, while a lower threshold means that even respondents who show only a slight tendency to inattentive responses are judged as C/IERs.

Ward and Meade (2023) proposed three levels of C/IER screening: minimal, moderate, and extensive. Since their proposal is to use a different detection index for each level, it does not directly correspond to the inattentive respondent probability produced by machine learning in this study, where DQS is used to define the outcome. However, multiple detection levels can be set by changing the threshold value used to identify C/IERs.

## Results

Table 4 shows the detection results for the PC responses when the model is applied to the test data. Table 5 shows the detection results for the smartphone responses. The results are given separately for the random forests and boosting cases. The row for each survey (labeled 1 through 13 in the case of PC responses and 1 through 16 in the case of smartphone responses) shows the C/IER detection results (accuracy, recall, precision, and balanced accuracy) when the test data are from the indicated survey and the training data are from all the other surveys. For example, in Table 4, the row for survey 4 indicates the degree to which respondents in survey 4 who did not comply with either of the two DQS items in the survey can be detected by the machine learning model developed with survey data from all the surveys except survey 4 (i.e., surveys 1–3 and 5–13). Two averages, Mean 1 and Mean 2, are shown in the bottom row of Tables 4 and 5. Mean 1 is the average of the values shown in the tables, while Mean 2 is the recalculated average using the data from all 13 (16) surveys. For example, the accuracy of Mean 2 is the rate at which 0,1 for all test data and 0,1 for the machine learning model are matched. As can be seen in the tables, there is little difference between Mean 1 and Mean 2.

**Table 4:** Detection results for the PC responses when criteria are 50%

	Boosting					Random forests				
ID	ACC	RCL	PRC	BAL	SPE	ACC	RCL	PRC	BAL	SPE
1	.67	.72	.71	.66	.60	.70	.73	.74	.69	.66
2	.74	.74	.50	.74	.74	.69	.80	.46	.73	.65
3	.77	.79	.69	.78	.76	.79	.77	.72	.78	.80
4	.82	.66	.67	.77	.88	.84	.68	.70	.79	.89
5	.73	.74	.72	.73	.71	.74	.73	.75	.74	.75
6	.65	.55	.70	.65	.75	.65	.56	.71	.66	.75
7	.72	.52	.58	.67	.81	.72	.53	.57	.67	.81
8	.74	.71	.63	.73	.76	.77	.77	.67	.77	.78
9	.71	.56	.58	.67	.78	.74	.58	.63	.70	.82
10	.68	.75	.57	.69	.63	.72	.80	.60	.73	.66
11	.77	.69	.74	.76	.81	.78	.69	.76	.77	.85
12	.56	.78	.45	.61	.44	.59	.84	.48	.64	.45
13	.72	.85	.66	.73	.61	.73	.86	.66	.73	.61
Mean 1	.71	.70	.63	.71	.71	.73	.72	.65	.72	.73
Mean 2	.72	.70	.62	.71	.73	.73	.72	.63	.73	.74
10.5%	.71	.70	.31	.71	.72	.73	.72	.33	.72	.73
50%	.71	.70	.69	.71	.72	.73	.72	.70	.72	.73
Old	.71	.69	.64	.70	.72	.68	.66	.60	.68	.69

ACC = accuracy, RCL = recall, PRC = precision, BAL = balanced accuracy, SPE = specificity, 10.5% is the mean when the C/IER ratio is artificially set to 10.5%, and 50% is the mean when the C/IER ratio is artificially set to 50%, Old is the result using only time, LS, maha, and maha_p as predictors

**Table 5:** Detection results for the smartphone responses when criteria are 50%

	Boosting					Random forests				
ID	ACC	RCL	PRC	BAL	SPE	ACC	RCL	PRC	BAL	SPE
1	.68	.57	.68	.67	.77	.73	.64	.74	.73	.81
2	.65	.76	.38	.69	.62	.58	.81	.33	.66	.51
3	.74	.83	.62	.76	.69	.75	.76	.66	.75	.75
4	.81	.65	.71	.77	.88	.80	.66	.69	.76	.87
5	.74	.80	.64	.75	.70	.74	.79	.64	.75	.71
6	.67	.63	.63	.66	.70	.67	.61	.63	.66	.71
7	.70	.70	.51	.70	.70	.73	.70	.55	.72	.74
8	.75	.80	.67	.76	.71	.78	.83	.71	.79	.75
9	.74	.67	.66	.73	.77	.73	.69	.64	.72	.76
10	.71	.67	.60	.70	.73	.74	.72	.63	.73	.75
11	.72	.71	.69	.72	.73	.74	.68	.74	.73	.79
12	.62	.81	.47	.67	.52	.63	.85	.48	.68	.52
13	.67	.89	.63	.66	.43	.72	.86	.68	.71	.56
14	.65	.84	.61	.66	.48	.65	.78	.61	.65	.51
15	.74	.53	.55	.68	.83	.79	.71	.62	.77	.82
16	.81	.76	.76	.80	.84	.83	.72	.82	.81	.89
Mean 1	.71	.73	.61	.71	.69	.73	.74	.64	.73	.72
Mean 2	.71	.72	.60	.72	.71	.73	.73	.62	.73	.72
10.5%	.72	.73	.31	.71	.69	.73	.74	.33	.73	.72
50%	.71	.73	.67	.71	.69	.73	.74	.69	.73	.72
Old	.73	.72	.65	.73	.74	.69	.69	.60	.70	.70

ACC = accuracy, RCL = recall, PRC = precision, BAL = balanced accuracy, SPE = specificity, 10.5% is the mean when the C/IER ratio is artificially set to 10.5%, and 50% is the mean when the C/IER ratio is artificially set to 50%, Old is the result using only time, LS, maha, and maha_p as predictors

The accuracy and precision values shown in Tables 4 and 5 are not strictly comparable across surveys, nor are the values from previous studies comparable. This is because the C/IER ratio differs between surveys and also differs from previous studies. Therefore, for accuracy, we obtained the balanced accuracy, which is a measure of unbalanced binary classification. Furthermore, the C/IER rates in the test data were 10% in Schroeders et al. (2022), 11% in Ozaki and Suzuki (2019), and 50% in Gogami et al. (2021). Therefore, to compare with previous studies on accuracy and precision, Tables 4 and 5 also show the mean values when the C/IER rate is artificially set to 10.5% and 50%, respectively. For example, setting the C/IER rate at 10.5% was achieved by artificially reducing the number of respondents whose outcome was C/IER, while leaving the cases where the outcome was attentively unchanged. Tables 4 and 5 show that accuracy, recall, precision, and balanced accuracy are slightly higher for random forests than boosting, so the results for random forests will be interpreted hereafter.

The row labeled “Old” in Tables 4 and 5 shows the results of a machine learning model using only time, LS, maha, and maha_p as predictors, which have been used in previous studies. Therefore, the difference between Old and Mean 1 indicates the effectiveness of the new predictor in this study.

### Comparison of results with previous studies

Table 6 summarizes the results of a comparison with previous studies. When comparing with Schroeders et al. (2022) and Ozaki and Suzuki (2019), the 10.5% case in Table 4 (PC response) is referenced; when comparing with Gogami et al. (2021), the 50% case in Table 5 (smartphone response) is referenced.

**Table 6:** Comparison with previous studies

	Device	C/IERs in test	C/IERs in overall	Sample overall	Sample in training	ACC	RCL	PRE	BAL	SPE
Schroeders et al. (2022)	PC	10%	40.3%	605	425	70%	60%	19%	66%	71%
Ozaki and Suzuki (2019)	PC	11%	31%	2000	1000	81%	66%	32%	74%	83%
Present study (10.5%)	PC	10.5%	39.7%	9308	4704	73%	72%	33%	72%	73%
Gogami et al. (2021)	Phone	50%	5%	4940	493	86%	86%	86%	-	-
Present study (50%)	Phone	50.0%	38.2%	12,536	5610	73%	74%	69%	73%	72%

ACC = accuracy, RCL = recall, PRC = precision, BAL = balanced accuracy, SPE = specificity

Comparing the results of Schroeders et al. (2022) to those of the present study, the present method is 3 points better in accuracy, 12 points better in recall, 14 points better in precision, 6 points better in balanced accuracy, and 2 points better in specificity. Thus, compared to the results of Schroeders et al. (2022), the detection accuracy is improved in all aspects. In particular, precision and recall are improved, which means that the probability of detecting an actual C/IER as a C/IER and the probability that a predicted C/IER is actually a C/IER are higher with the developed method.

Comparing the results of Ozaki and Suzuki (2019) to those of the present study, the results of this study are inferior in accuracy by 8 points, superior in recall by 6 points, almost the same results in precision, slightly inferior in balanced accuracy, and 10 points inferior in specificity. Thus, compared to Ozaki and Suzuki (2019), the probability of detecting an actual C/IER as a C/IER is increased, but the probability of detecting an actual attentive respondent as an attentive respondent is decreased.

The accuracy, precision, and recall reported by Gogami et al. (2021) were each approximately 86%, meaning that the results of the present study are inferior in all three aspects. It is important to note, however, that the three previous studies, including Gogami et al. (2021), used test data from surveys with the same content as the training data. On the other hand, the results of the present study were produced using training data and test data from questionnaires whose content was quite different. The fact that the proposed method was able to achieve higher accuracy than two of the three previous studies in some accuracy indices can thus be considered a notable advance in establishing the generalizability of the method using machine learning. The reason for the higher accuracy than existing methods is that multiple survey data were treated in an integrated manner, as shown in Fig. 2, which resulted in the sample size of the training data being much larger than in previous studies, as shown in Table 6.

It is also worth mentioning that since Gogami et al. (2021) used smartphone response data, the results of the present study are the best ever obtained for PC response data for recall. It is worth noting, too, that the superiority of Gogami et al. (2021) in detection accuracy might be attributable to its use of smartphone screen operation data (the number of times text was deleted and the respondent’s scrolling speed, etc.). Since this information was not collected in the 16 surveys, incorporating it into future studies is an issue to be considered.

### Effectiveness of the new predictor

The difference between Old and Mean 1 in Tables 4 and 5 shows the effect of adding a new predictor to the model with time, LS, maha, and maha_p. In the case of boosting, there is little difference and almost no effect of adding a new predictor. In fact, the PRC in Table 5 is about 4% lower when new predictors are included, suggesting that the generalization performance of the model may be reduced. On the other hand, in the case of random forests, the effect of adding a new predictor is about 2% to 5% for each indicator, indicating that the inclusion of a new predictor is effective, although not large.

### Predictor importance

Before showing the predictor importance, the correlation matrix between predictors calculated using all the data is shown in Table 7. What is striking about this correlation matrix is that there is almost no correlation between response time and the other predictors. Although not shown in the paper, a similar trend was observed when the correlation matrix was calculated for each survey: In four of the 13 surveys, the correlation between time and LS was more negative than – .10 for PC respondents. This is similar to the correlation between time and LS of – .05 in Maniaci and Rogge (2014) and – .12 in Meade and Craig (2012). This suggests that although one would think that shorter response times would lead to larger LS values, this is not the case. This suggests the importance of using other predictors in combination with response time. The correlation between LS and maha is – .41, which is significantly different from – .15 in Maniaci and Rogge (2014) and .10 in Meade and Craig (2012). This can be interpreted as a result of the smaller maha of respondents who answered consecutively in the intermediate category (i.e., longer LS), since, as mentioned earlier, Japanese have a strong tendency to respond in the intermediate category and the mean of the variable tends to be the value assigned to the intermediate category.

**Table 7:** The correlation matrix between predictors

Predictor	1	2	3	4	5	6	7	8	9	10	11	12	13
1. time	-	– .02	– .01	– .01	.01	– .01	.01	.00	.00	.00	.00	.02	.02
2. LS	– .03	-	.72	.42	– .38	.50	– .11	– .58	– .18	.27	– .34	– .12	– .11
3. R2C	– .03	.73	-	.45	– .38	.54	– .11	– .39	– .18	.25	– .20	– .12	– .15
4. R3C	– .03	.51	.58	-	– .89	.52	– .62	– .03	– .52	.54	.02	– .05	– .05
5. AC	.01	– .67	– .46	– .23	-	– .43	.62	.00	.60	– .52	– .03	.04	.04
6. MAC	– .01	– .41	– .28	– .13	.79	-	– .20	– .16	– .23	.26	– .06	– .15	– .22
7. maha	.03	– .41	– .40	– .42	.11	.07	-	– .21	.53	– .61	– .16	.08	.04
8. maha_p	– .03	.47	.49	.51	– .15	– .07	– .86	-	– .04	– .02	.79	.13	.11
9. maha2	.01	– .22	– .19	– .22	.02	.01	.62	– .51	-	– .79	– .03	.02	.02
10. maha2_p	– .01	.34	.27	.29	– .14	– .08	– .53	.57	– .74	-	.01	– .03	– .01
11. absdevi	.00	– .20	– .18	– .24	– .11	– .09	.65	– .66	.53	– .64	-	.10	.09
12. time_m	– .02	– .20	– .18	– .16	.13	.09	.07	– .11	.04	– .09	.16	-	.86
13. nitems	– .02	– .14	– .20	– .24	.10	.08	.06	– .09	.03	– .06	.10	.79	-

The lower triangular matrix represents the case of PC responses and the upper triangular matrix represents the case of smartphone responses. time is the number of seconds for response, LS is the maximum number of consecutive responses, R2C is the response limited to no more than 2 categories, R3C is the response limited to no more than 3 categories, AC is the number of consecutive responses to items in adjacent categories, MAC is the maximum number of consecutive responses to items in adjacent categories, maha is the Mahalanobis distance using 12 items, maha_p is the *p* value of Mahalanobis distance using 12 items, maha2 is the Mahalanobis distance using the two variables with highest correlation, maha2_p is the *p* value of Mahalanobis distance using the two variables with highest correlation, absdevi is the sum of absolute values of deviations from mean vector, time_m is the median total response time for each survey, nitems is the number of survey items

**Fig. 3:**
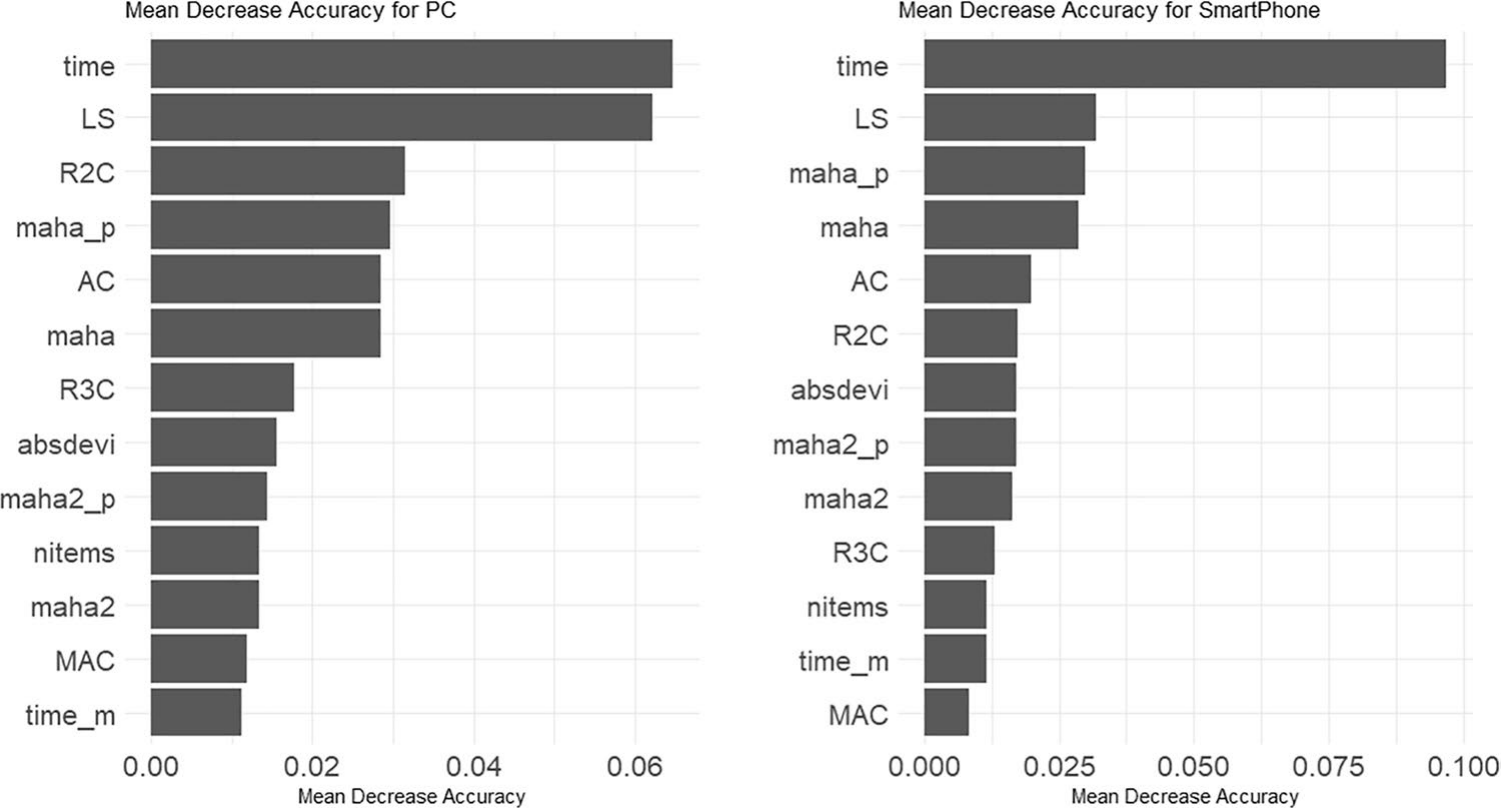
Predictor. *Note:* Mean accuracy decrease represents the amount of classification accuracy lost when each predictor value is shuffled among respondents; the larger the value, the more important is the predictor. times is the number of seconds for response, LS is the maximum number of consecutive responses, maha_p is the *p* value of Mahalanobis distance using 12 items, R2C is the response limited to no more than 2 categories, AC is the number of consecutive responses to items in adjacent categories, maha is the Mahalanobis distance using 12 items, R3C is the response limited to no more than 3 categories, absdevi is the sum of absolute values of deviations from mean vector, maha2_p is the *p* value of Mahalanobis distance using the two variables with highest correlation, nitems is the number of survey items, maha2 is the Mahalanobis distance using the two variables with highest correlation, MAC is the maximum number of consecutive responses to items in adjacent categories, time_m is the median total response time for each survey

The random forest model to detect DQS results was retrained using response data from all PC responses (*n* = 196 × 2 × 13 = 5096) and all smartphone responses (*n* = 187 × 2 × 16 = 5984). Figure 3 shows the mean decrease in accuracy as an indicator of predictor importance in the random forest model. The left side of Fig. 3 shows the PC responses; the right side shows the smartphone responses. Mean decrease in accuracy indicates how much classification accuracy is lost when each variable is excluded from the model: the larger the value, the more important the predictor.

As indicated in the figure, the response time is the most important factor for both the PC and smartphone responses, followed, in order, by LS, mama_p, and maha. The importance of response time has been noted by Leiner (2019), Ward and Meade (2023), and others. mama_p and maha decreased classification accuracy when one of them was kept and the other was deleted. Therefore, it was found that the effects of both predictors were seen even when they were entered into the predictor set at the same time.

Among the predictors not previously used in the context of C/IER detection, we found that including R2C, AC, maha2, maha2_p, absdevi, time_m, and nitems helped improve accuracy. These were found to be almost equally effective and less effective than maha_p and maha. R3C and MAC were also found to have the lowest effect among the predictors used. This is probably because R2C substitutes for the role of R3C and AC substitutes for MAC. However, the inclusion of R3C and MAC helped to improve accuracy and, like R2C, AC, maha2, maha2_p, and absdevi, are indicators that can be easily computed given Likert-scale data. In addition, as discussed above, AC and MAC can detect cases that cannot be detected by IRV, and therefore, are recommended for future use in studies of C/IER detection. For maha2 and maha2_p, it was found that, as with maha and maha_p, the inclusion of both at the same time improved the estimation accuracy.

The questionnaire-level predictors, time_m and nitems, are the first predictors used in this study and were found to be as effective as R2C and others. time_m and nitems are measures of the approximate response time for each survey and the length of the questionnaire. The results indicate that time_m and nitems have an effect that cannot be fully substituted by the response time at the individual level. Time_m and nitems should be included when developing machine learning models using multiple survey data, as in this study.

Overall, the results of this study support claims 1 through 6 regarding the advantages of the proposed method. However, the effectiveness of the new predictor was not found in boosting, but a small effectiveness was found in random forests. This indicates that the contribution of the new predictor to the development of a generic method was small, and that it was more effective to construct the model by integrating the multiple survey data as shown in Fig. 2. The transformation of the predictor values by the proposed method was also considered effective.

## Discussion

This study developed a generic method for detecting inattentive or careless survey respondents (C/IERs) using machine learning. This section summarizes the limitations of this study, future research directions, and recommendations for researchers using the methods proposed in this study.

## Limitations

This study had the following two limitations. First, because this study uses DQS for outcome, the predicted inattentive response probability represents the tendency of respondents to not comply with DQS and so is not an exhaustive method for detecting various types of inattentive respondents. This study used DQS for outcome because, as discussed in subsection “Outcome,” DQS is unambiguous in scoring (Ward & Meade, 2023), and previous studies have argued for the validity of DQS (Maniaci & Rogge, 2014; Ward & Meade, 2023).

Second, this method is only applicable when the questionnaire contains a Likert scale and response time data are available for each respondent. Also, this method works well when the Likert scale contains reverse-worded items that measure the same construct or items that measure different constructs. This is due to the use of predictors such as LS.

## Future research directions

A number of research directions should be noted here. The first is regarding improving accuracy. The results in Tables 4, 5, and 6 are partially superior to those of Ozaki and Suzuki (2019) and Schroeders et al. (2022), but fall short of those of Gogami et al. (2021), which used smartphone operation information. Since smartphone operation information is not available for some surveys, it is significant that this study showed that even in the absence of smartphone operation information, it is possible to obtain high prediction accuracy. On the other hand, Buchanan and Scofield (2018) also showed the effectiveness of using one of the operational data, the number of clicks during the response, to detect C/IERs. The inclusion of smartphone operation information and number of clicks as predictors is expected to improve accuracy.

Furthermore, although only 16 surveys were available for this study, if a larger number of surveys were available, models could be developed for each research category. This may also contribute to an improvement in accuracy. Recently, Yeung and Fernandes (2022) developed a method to extract invalid text sentences by machine learning. The same is possible with GPT-4. Although this study uses little information obtained from text sentences as predictors, it is expected that the accuracy of C/IER detection will be increased by using the evaluation values for text sentences as predictors.

The third point is to develop a machine learning model with outcomes other than DQS. By developing a machine learning model with other outcomes such as ARS, it may be possible to understand each respondent’s response behavior in more detail. The results of this study will help overcome the first limitation mentioned above.

The fourth point is to compare this study with a series of studies that modeled the response behavior of C/IERs and attentive respondents by a latent response mixture model (Ulitzsch, Pohl, et al., 2022a; Ulitzsch, Yildirim-Erbasli, et al., 2022b; Ulitzsch, Pohl, et al., 2023a; Ulitzsch, Shin, et al., 2023b). If response time per page or Likert scale data of different polarity is available, these previous studies can also be used as generically as this study, regardless of the content of the questionnaires. In particular, the model of Ulitzsch, Yildirim-Erbasli, et al. (2022b) allows us to examine inattentive response tendencies by respondent and by item. This can be rephrased as being able to detect fluctuations in respondents’ attention. Since the method developed in this study may also be able to detect fluctuations in respondents’ attention by changing the Likert scale items applied, a comparison from this perspective is also possible.

## Recommendations for researchers using the proposed methods

To actually use this method, it is first necessary to construct a machine learning model. To do so, it is necessary to collect data from multiple web surveys. It is also necessary that all surveys include DQS and Likert scales and that response time be measured.

When extracting predictors from the collected raw data, we can use the R code calculating_predictors.R as described in the Appendix. In developing a machine learning model using the training data containing the computed predictors and applying that model to the test data (survey data that were not used as training data), analysis.R can be used. analysis.R allows us to estimate the inattentive response probability for each respondent on the test data. If the prediction accuracy for the test data is high, a machine learning model can be developed using the data from all surveys. Note, however, that random sampling is desirable so that the ratio of C/IERs to attentive respondents is 50:50. The model can then be applied to any survey data to detect C/IERs. See the Appendix for the details of the R code.

It also seems necessary to create country-specific models when using this method. The model for this study was developed using data from Japanese respondents. Since the tendency to respond to survey items differs from country to country, it is not certain whether the model developed in this study will be applicable to data from respondents in other countries.

## Data Availability

The first author’s affiliation with the research ethics committee does not permit publication of the data in principle, so the data cannot be made public. However, sample data are available at https://osf.io/dx2mf. Results for varying the threshold from 0.1 to 0.9 in machine learning predictions are available at https://osf.io/2t64w. **Code availability** (software application or custom code) The R codes for calculating predictors and for machine learning predictions are available at https://osf.io/dx2mf. Appendix provides examples of analyses using the sample data and analysis code.

## Appendix

### Example data and codes

#### Purpose of appendix and example data and R codes

In this Appendix, sample data and sample R codes are presented. Three sample data sets are available, all of which were artificially generated. The sample size for each sample data is 500. These are called data1, data2, and data3. data1 and data2 are combined as the training data and data3 is used as the test data. Sample data and sample R codes are available at https://osf.io/dx2mf.

All three surveys include ID in the first column, response time (restime) in the second column, and whether the respondent correctly responded to the DQS (= 0) or not (= 1) in the last column. All surveys also include Likert scales; the 12 items used to calculate LS and other predictors are Q78 to Q89 in data1, Q86 to Q97 in data2, and Q73 to Q84 in data3. The Likert scale used is not limited to 12 items and can differ between surveys. Because the data are artificial, there are no specific item contents for each survey instrument.

R code calculating_predictors.R is used to read the data and extract the variables necessary to develop the inattentive respondent detection model from the data. The order of the extracted variables is ID, time, time_m, LS, AC, MAC, R2C, R3C, absdevi, maha, maha_p, maha2, maha2_p, nitems, DQS. Line 8, datalikert<-c("Q78", "Q89"), is for data1, so if you want to run it for data2, you need to read data2 on line 4 and then datalikert<-c("Q86", "Q97"). The same is true for data3. If you apply this R code to your own data, please change this part of the code. In this example code, the extracted data set is data1_analysis for data1, data2_analysis for data2, and data3_analysis for data3.

R code analysis.R combines data1_analysis and data2_analysis, develops a machine learning model for inattentive respondent detection using the combined data, and applies the model to data3_analysis. If the number of data is not three, change this R code accordingly. The outputs are accuracy, recall, precision, and balanced accuracy when applied to data3_analysis.

The minimum number of inattentive and non-inattentive respondents in data1_analysis and data2_analysis (which is 211 in the sample data) is obtained, and then inattentive and non-inattentive respondents from data1_analysis and data2_analysis are randomly selected to achieve the minimum for each. In this way, the numbers of inattentive and non-inattentive respondents are the same. This is expected to improve the prediction accuracy.

### Results

Accuracy is obtained at line 84, recall at line 86, precision at line 89, balanced accuracy at line 92, and specificity at line 96.
